# Added Value of Impact Microindentation in the Evaluation of Bone Fragility: A Systematic Review of the Literature

**DOI:** 10.3389/fendo.2020.00015

**Published:** 2020-02-07

**Authors:** Manuela Schoeb, Neveen A. T. Hamdy, Frank Malgo, Elizabeth M. Winter, Natasha M. Appelman-Dijkstra

**Affiliations:** Department of Medicine, Division of Endocrinology and Center for Bone Quality, Leiden University Medical Center, Leiden, Netherlands

**Keywords:** fracture risk, osteoporosis primary and secondary, rare bone diseases, bone quality, bone material strength index (BMSi), osteoprobe, bone mineral density, dual energy x-ray absorptiometry (DXA)

## Abstract

The current gold standard for the diagnosis of osteoporosis and the prediction of fracture risk is the measurement of bone mineral density (BMD) using dual energy x-ray absorptiometry (DXA). A low BMD is clearly associated with increased fracture risk, but BMD is not the only determinant of bone strength, particularly in secondary osteoporosis and metabolic bone disorders in which components other than BMD are affected and DXA often underestimates true fracture risk. Material properties of bone which significantly contribute to bone strength have become evaluable *in vivo* with the impact microindentation (IMI) technique using the OsteoProbe® device. The question arises whether this new tool is of added value in the evaluation of bone fragility. To this effect, we conducted a systematic review of all clinical studies using IMI *in vivo* in humans also addressing practical aspects of the technique and differences in study design, which may impact outcome. Search data generated 38 studies showing that IMI can identify patients with primary osteoporosis and fractures, patients with secondary osteoporosis due to various underlying systemic disorders, and scarce longitudinal data also show that this tool can detect changes in bone material strength index (BMSi), following bone-modifying therapy including use of corticosteroids. However, this main outcome parameter was not always concordant between studies. This systematic review also identified a number of factors that impact on BMSi outcome. These include subject- and disease-related factors such as the relationship between BMSi and age, geographical region and the presence of fractures, and technique- and operator-related factors. Taken together, findings from this systematic review confirm the added value of IMI for the evaluation and follow-up of elements of bone fragility, particularly in secondary osteoporosis. Notwithstanding, the high variability of BMSi outcome between studies calls for age-dependent reference values, and for the harmonization of study protocols. Prospective multicenter trials using standard operating procedures are required to establish the value of IMI in the prediction of future fracture risk, before this technique is introduced in routine clinical practice.

## Introduction

Bone fragility is complex and its evaluation represents a significant challenge in clinical practice. The tools used to assess bone strength and thus fracture risk have so far included the measurement of bone mineral density using dual energy x-ray absorptiometry (DXA) and the evaluation of clinical risk factors for increased bone fragility using the FRAX algorithm. BMD measurements have been routinely performed in the clinic for over three decades and experience with their use is substantial. A low BMD has been clearly associated with increased fracture risk, but evidence has been accumulating over the past decade for factors contributing to bone strength other than BMD, as only one third of an individual's fracture risk is being explained by BMD values (1). The strength of bone thus not only depends on bone mineral density but also on its architecture at the macro-, micro- and nanolevel and on its material composition (2). Available tools for the evaluation of these various components of bone strength have so far included primarily histological evaluation of bone biopsies to assess bone histomorphometry parameters and nanoindentation to assess material properties of bone. Other tools more recently included high resolution peripheral quantitative computed tomography (HR-pQCT) to assess bone structure, and finite element analysis (3, 4). However, these methods are either invasive and time-consuming in their analysis, or associated with high radiation exposure. Material properties of bone, which significantly contribute to bone strength could until recently only be assessed *ex vivo* on a transiliac bone biopsy specimen. Since the introduction of Reference Point Indentation, the possibility has emerged for directly evaluating tissue-level properties of bone in humans *in vivo* (5, 6).

Impact microindentation (IMI) using the handheld OsteoProbe® device has been developed for use in the clinic as an adaption of the original Reference Point Indentation technique (7). Experience has been accumulating with the use of this technique and data have been collected from an increasing number of patients mainly with primary or secondary osteoporosis. A standard operating procedure for IMI has also been recently published to harmonize collection of data (8). Although results have been so far promising, outcomes have not always been concordant between studies or centers so that the added value of this technique in the evaluation of bone fragility still remains to be established. To address this issue, we conducted a systematic review of the literature of all clinical studies in which impact microindentation was performed *in vivo* in humans using the OsteoProbe® device including those published as meeting abstracts. Our objective was to assess the potential added value of impact microindentation in the evaluation of fracture risk in clinical practice. In this process, we also reviewed available literature on practical aspects of the technique and on differences in study design, which may explain differences in outcome between studies, adding new data to those studies published in the last review on the topic in 2017 (9).

### The Reference Point Indentation Technique

Reference Point Indentation (RPI) is a technique which enables the assessment of material properties of bone by indenting the bone surface of the tibia *in vivo* in humans. The principle of RPI is based on the hypothesis that indentation of the bone surface results in separation of mineralized collagen microfibers, resulting in microcracks (10). The observation that RPI induced microcracks similar to those observed in fractured cadaveric human bone samples led to the development of the technique *in vivo* in humans in 2006 with a view to assessing the ability of bone to resist fractures. Two Reference Point Indentation devices have so far been used. The original Biodent™ device has been used in the laboratory in animal studies, on human cadaveric bone, and also in early studies *in vivo* in humans (10, 11). The handheld device OsteoProbe® was adapted from the Biodent™ device for *in vivo* use in large animals and in the clinic (7). To avoid confusion in the interpretation of data, a different nomenclature has been proposed for the two devices in 2016 using the term cyclic reference point microindentation (CMI) for the Biodent™ device, and impact microindentation (IMI) for the OsteoProbe® device (8). Since the OsteoProbe® is currently the only tool used in the clinic, and there have been no new publications using the Biodent™ *in vivo* in humans since 2013, with all prior publications included in the last review (9), this systematic review of the impact microindentation technique covers published literature of studies only using the OsteoProbe®.

**Figure 1:**
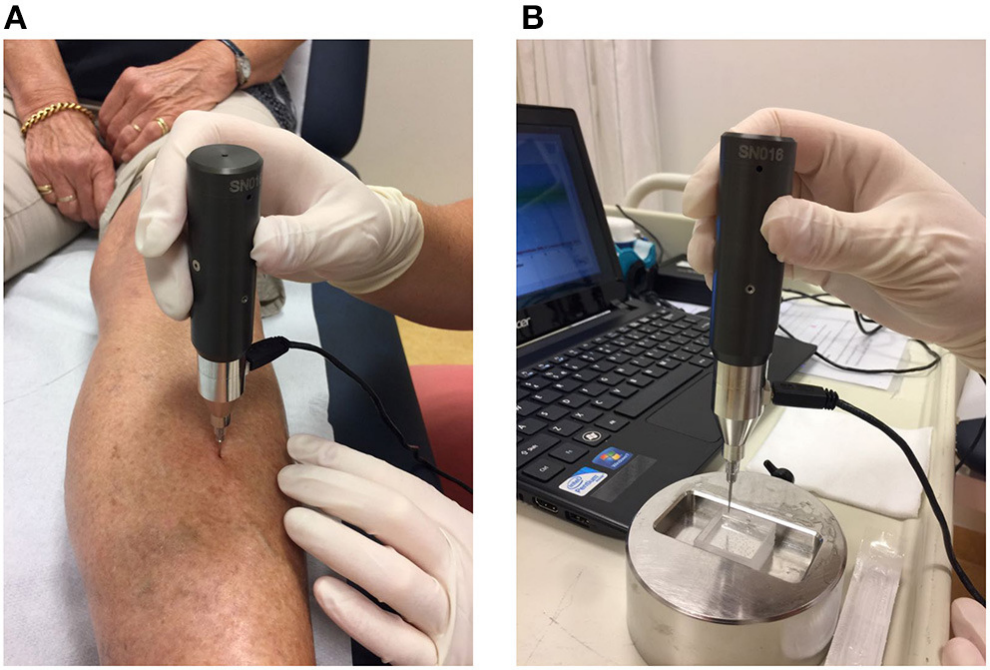
**(A)** Method of use of the OsteoProbe® on the midshaft of the tibia after the application of a local anesthetic, **(B)** measurement performed on the polymethylmethacrylate (PMMA) reference phantom.

Using the OsteoProbe®, measurements are performed at a localization at the mid-shaft of the tibia after applying local anesthetic with the patient in the supine position (Figure 1A). The probe is gently inserted in the skin at the point of interest until the bone surface is reached, following which the cortical bone of the tibia is indented by an impact delivered by the OsteoProbe®. The resistance of bone tissue to this applied mechanical challenge is expressed as the measured distance covered by the test probe after impact of the probe into bone: the Indentation Distance Increase (IDI). Poorly performed measurements, usually due either to slipping of the test probe on the surface of the bone or to unintentional moving of the subject's leg, are discarded. After 10 adequate measurements are obtained, five further measurements are performed on a polymethylmethacrylate (PMMA) reference phantom (Figure 1B). The software then calculates the outcome parameter of IMI: the Bone Material Strength index (BMSi). BMSi is defined as 100 times the harmonic mean of IDI from impact into the PMMA phantom divided by the average IDI from impact into bone tissue (7). The lower bone strength is, the deeper the probe indents the bone surface (high IDI) and the lower is the outcome parameter BMSi.

## Methods

### Search Strategy and Eligibility Criteria

A search strategy was designed with the help of an experienced librarian for the systematic review of the literature on impact microindentation. The search, which was conducted in PubMed, EMBASE and Web of Science, included all original published articles and meeting abstracts in the English language, updated up to August 2019. Relevant keywords were used, including free text words (see Supplementary Material for details of complete search). Studies using the cyclic RPI technique with the pre-clinical device Biodent™, and studies using early OsteoProbe I™ and II™ devices were excluded from analyses. All articles identified by the search were assessed by two independent investigators.

### Search Data Extraction

Studies were independently reviewed by the two independent investigators and the following data were extracted: (1) Demographic data on patient and control populations (clinical characteristics, population size); (2) outcome data (BMSi and BMD at the femoral neck (FN)); (3) Factors potentially influencing BMSi outcome (age, gender, body mass index (BMI), geographical region, prevalent fractures, BMD, number of operators, intra- and interobserver coefficient of variation, number of indentations per BMSi obtained); (4) potential complications arising from IMI. Extracted data were reported as presented in the articles, as mean ± SD, mean (SEM), or median (IQR).

### Quality Assessment

The quality of full publications was independently assessed using the Newcastle-Ottawa (NO) Scale, which includes three domains: selection, comparability and exposure/outcome and was adapted for this review (see Supplementary Table). Based on the total quality score, articles were scored out of a maximum score of ten as unsatisfactory (0–4), satisfactory (5–7), or good (8–10).

## Results

### Study Selection

**Figure 2:**
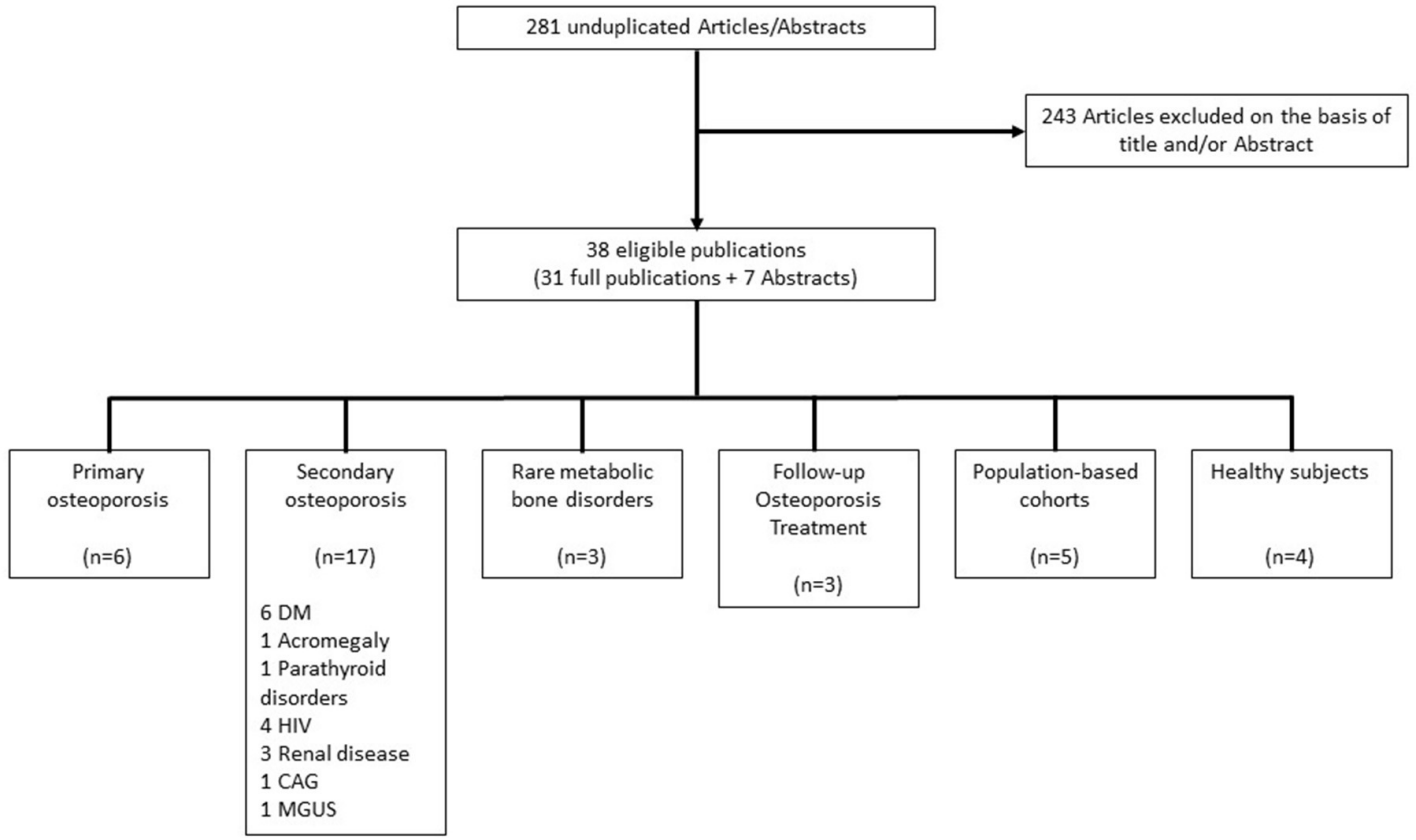
Flowchart of study selection. DM, Diabetes mellitus; HIV, Human immunodeficiency virus; CAG, Chronic atrophic gastritis; MGUS, Monoclonal gammopathy of undetermined significance.

The search yielded 456 articles and 102 meeting abstracts. Two hundred and seventy-seven duplicate articles in ≥2 electronic data bases were excluded, and 281 unique studies were further assessed; (Figure 2). Two hundred and forty-three of these were excluded on the basis of title and abstract: 218 studies were not using the OsteoProbe®, eight were using the OsteoProbe® either in animal studies (12–14) or in *ex vivo* human bone (15–19), two described primarily technical aspects of RPI devices (8, 20) and 15 were review articles, eight describing different techniques for the assessment of bone quality including RPI (21–28), and seven specifically on RPI (7, 9, 20, 29–32). Included in the final evaluation were 31 original articles and seven studies published only as meeting abstracts using the OsteoProbe® microindentation device *in vivo* in humans.

### Quality Assessment

Based on the adapted Newcastle-Ottawa Scale (adapted NO Scale: Supplementary Table), methodological quality of studies was good in eight studies (33–40), satisfactory in 15 studies (41–55), and unsatisfactory in six studies (56–61) (Tables 1–**6**). The NO Scale could not be used for the evaluation of two fully published studies (67, 69) and for all meeting abstracts (62–66, 68, 70) because of incomplete data. Of the 31 full articles, 8 (25.8%) were thus of unsatisfactory methodological quality or unevaluable. Notwithstanding, for the purpose of completeness of this systematic review of the literature we report on all full publications as well as meeting abstracts yielded by our search strategy reporting the use of IMI *in vivo* in humans.

**Table 1:** Impact microindentation studies in subjects with osteoporosis/fractures.

**References**	**Subjects**	**(m/f)**	**Age (years)**	**FN BMD (g/cm^**2**^)**	**BMSi**	**Relationship of BMSi with**			**Study quality scale (0–10)**
						**Age**	**BMD**	**Fx**	
Malgo et al. (33)	Fx	63 (24/39)	62.6 ± 9.6 (40–85)	0.67 ± 0.09	79.9 (0.6) (78.7–81.1)	Neg	No	Pos	8
	No Fx	27 (13/14)	57.1 ± 9.5 (40–85)	0.69 ± 0.08	82.4 (1.0) (80.3–84.5)				
Malgo et al. (34)	NVF only	53 (14/39)	62.8 ± 8.3 (40–85)	0.65 ± 0.07	78.9 (0.7) (77.5–80.3)	Neg	No	Pos	9
	VF + NVF	34 (14/20)	62.8 ± 9.9 (40–85)	0.69 ± 0.09	78.3 (0.9) (76.5–80.1)				
	VF only	14 (8/6)	64.7 ± 9.3 (40–85)	0.70 ± 0.09	78.4 (1.4) (75.4–81.4)				
	No Fx	31 (11/20)	57.5 ± 9.9 (40–85)	0.68 ± 0.07	82.5 (0.9) (80.7–84.3)				
Duarte Sosa and Fink Eriksen (38)	Stress Fx	30 (0/30)	39.0 ± 13.9 (19–64)	0.92 ± 0.25	70.5 ± 8.7 (67.4–73.6)	No	No	Pos	8
	No Fx	30 (0/30)	42.3 ± 9.8 (23–66)	1.05 ± 0.11	77.1 ± 7.2 (74.5–79.7)				
Sosa and Eriksen (35)	NH/NVF	17 (0/17)	66.2 ± 9.1 (50–85)	0.77 ± 0.07	73.1 ± 6.5 (69.8–76.4)	No	No	Pos	10
	HF	25 (0/25)	68.2 ± 10.0 (50–85)	0.76 ± 0.11	72.0 ± 6.5 (69.3–74.7)				
	VF	24 (0/24)	67.8 ± 10.1 (50–85)	0.71 ± 0.10	70.1 ± 7.1 (67.2–73.0)				
	No Fx	66 (0/66)	66.5 ± 7.9 (50–85)	1.02 ± 0.13	76.4 ± 6.2 (74.9–77.9)				
Rozental et al. (50)	DRF	57 (0/57)	64.2 ± 10.5 (>50)	0.68 ± 0.11	74.4 ± 8.8 (72.1–76.7)	Neg	Pos	DRF Pos HF No	6
	HF	42 (0/42)	75.7 ± 10.9 (>50)	0.61 ± 0.12	74.6 ± 8.5 (72.0–77.2)				
	No Fx	93 (0/93)	67.3 ± 7.6 (>50)	0.72 ± 0.10	77.4 ± 8.8 (75.6–79.2)				
Popp et al. (55)	AFF	15 (0/15)	71.8 ± 10.8	0.69 ± 0.03	76.5 ± 10.9 (70.5–82.5)	No	No	No	5
	HF	20 (0/20)	74.6 ± 8.3	0.62 ± 0.03	78.3 ± 9.3 (73.9–82.7)				
	BP >5 years	30 (0/30)	71.9 ± 9.1	0.60 ± 0.19	76.6 ± 10.5 (72.8–80.4)				
	BP–naive	88 (0/88)	65.9 ± 5.6	0.71 ± 0.01	80.1 ± 8.3 (78.4–81.8)				

*Age is presented as mean ± SD (range, if reported), BMD is presented as mean ± SD and BMSi is presented as mean ± SD (95% CI) or mean (SE) (95% CI). 95% CI calculated from data provided in the publication. AFF, Atypical femoral fracture; BMD, Bone mineral density; BMSi, Bone material strength index; BP, Bisphosphonate; DRF, Distal radius fracture; FN, Femoral neck; Fx, Fracture; HF, Hip fracture; NH/NVF, Non-hip non-vertebral fracture; NVF, Non-vertebral fracture; VF, Vertebralfracture*.

### Characteristics of Studies Generated by the Search

Of the 38 reported studies using the OsteoProbe®, 17 included patient groups with a known increased risk for fragility fractures on the basis of secondary osteoporosis, although this was not fully reflected by BMD values (36, 41, 45–49, 52, 53, 56, 58, 59, 62–66). The majority of these studies included patients with endocrine disorders such as diabetes mellitus and acromegaly (*n* = 8) (36, 41, 48, 49, 62–65). Six studies focused on patients having sustained fragility or stress fractures (33–35, 38, 50, 55), three studies evaluated BMSi outcomes in patients receiving osteoporosis treatment (37, 44, 68) and another three studies reported on patients with a rare metabolic bone disorder: Type 1 Gaucher Disease, Paget's and Camurati-Engelmann disease (57, 60, 67). Seven studies, two of which included patients with diabetes mellitus (41, 63), were performed in two population-based cohorts from Sweden and Australia, both designed to assess epidemiological data in osteoporosis (39, 43, 51, 54, 69). Four other studies included healthy individuals (40, 42, 61, 70) (Figure 2).

The number of patients in whom BMSi was measured varied greatly between studies, with the smallest study including only seven subjects (68) and the largest 489 subjects (41). All studies were performed in adults with ages ranging from a median of 33.9 years (27.6–53.8 years) (46) to a mean of 78.3 ± 1.1 years (51). Fourteen studies included only women (35, 37–43, 48–51, 55, 68), and four studies included only men (54, 63, 64, 69). Although not always explicitly stated, 17 studies appear to have overlapping patient or control cohorts (33, 36, 39, 41, 43, 45, 51–54, 57–60, 63, 67, 69).

### Impact Microindentation Studies in Patients with Fractures (Table 1)

Initial studies using IMI aimed at establishing the value of the then novel technique in the evaluation of bone fragility in patients who had sustained a fracture or were at high risk of sustaining one. Six of these studies evaluated the association between BMSi and prevalent fractures (33–35, 38, 50, 55).

Confirming data initially obtained by the Spanish group using the earlier device Biodent™ (10, 11), our group demonstrated that in the presence of comparable BMD values, BMSi was lower in 63 patients who had sustained a fragility fracture compared to 27 patients who had not [respectively, 79.9 (SE 0.6) vs. 82.4 (SE 1.0), *p* = 0.032]. BMSi was also comparable in patients with fragility fractures irrespective of whether they had osteopenia or osteoporosis (33). A subsequent study conducted in 132 patients, including data from the 90 patients from the original publication, confirmed that BMSi was lower in patients with fragility fractures (*n* = 101) compared to those who never sustained a fracture (*n* = 31) [respectively, 79.0 (SE 0.5) vs. 82.5 (SE 0.9), *p* = 0.001], independently of BMD measurements. Interestingly we also observed that measured BMSi values were comparable in fracture patients, regardless of type of fracture: non-vertebral (*n* = 53) [BMSi 78.9 (SE 0.7)], vertebral (*n* = 14) [BMSi 78.4 (SE 1.4)] or combined non-vertebral and vertebral fractures (*n* = 34) [BMSi 78.3 (SE 0.9)] (34).

Duarte Sosa et al. confirmed these findings by demonstrating that 66 postmenopausal women with osteoporosis and fragility fractures had significantly lower BMSi than age-matched postmenopausal women who had normal BMD values and no fractures (respectively, 71.5 ± 7.3 vs. 76.4 ± 6.2, *p* = 0.008) (35). Also in keeping with results from our group (34) there was no difference in BMSi whether patients had vertebral fractures (*n* = 24), hip fractures (*n* = 25) or non-hip non-vertebral fractures (*n* = 17), with BMSi 70.1 ± 7.1 vs. 72.0 ± 6.5 vs. 73.1 ± 6.5, respectively (35). Findings from this study also demonstrated that BMSi was inversely related to severity of vertebral fractures as evaluated by Genant's grading score in the 24 patients with vertebral fractures (*r*^2^ = 0.19, *p* < 0.05), although these results could not be reproduced by our group or by that of Rudäng et al. (34, 43). Another study from the Norwegian group excluding patients with osteoporosis or previous low-energy fractures, but including women with stress fractures of the lower extremities or pelvis, demonstrated significantly lower BMSi in 30 patients with stress fractures (BMSi 70.5 ± 8.7), than in controls without fractures (BMSi 77.1 ± 7.2, *p* = 0.01) (38).

In a cohort of 192 postmenopausal women, Rozental et al. showed that BMSi was lower in addition to lower BMD at the FN and LS in 57 patients with distal radius fractures than in 93 fracture-free controls (BMSi, respectively, 74.4 ± 8.8 vs. 77.4 ± 8.8, *p* = 0.04) (50). This was also the case for the 42 patients with hip fractures although this did not reach statistical significance compared to controls (BMSi 74.6 ± 8.5, *p* = 0.09).

In a study addressing whether cortical tissue properties of bone are altered in atypical femoral fractures (AFF), BMSi was measured in 15 postmenopausal women with this rare fracture. In these patients, BMSi was lower, albeit not significantly, compared to that in 20 patients with hip fractures, 30 long-term bisphosphonate users, and 88 patients never treated with antiresorptives (BMSi 76.5 ± 10.9 vs. 78.3 ± 9.3 vs. 76.6 ± 10.5 vs. 80.1 ± 8.3, respectively) (55).

In summary, BMSi was found to be lower in all patients with fragility fractures compared to non-fracture controls independently of site of fracture and generally independently of BMD values, suggesting that tissue material properties of bone are altered in fragility fracture patients and that BMSi measured at the tibia is associated with increased bone fragility at all relevant skeletal sites.

### Impact Microindentation Studies in Secondary Osteoporosis (Table 2)

Any systemic disorder may affect the skeleton and alter the material properties of bone thus increasing fracture risk. Our literature search yielded 17 studies addressing the value of IMI in assessing fracture risk in secondary osteoporosis: eight in patients with a variety of endocrine diseases (36, 41, 48, 49, 62–65), four in patients infected with the Human Immunodeficiency Virus (HIV) (46, 47, 52, 59), three in patients with chronic kidney disease (45, 53, 58), one in patients with chronic atrophic gastritis (56) and one in patients with monoclonal gammopathy of undetermined significance (MGUS) (66).

Table 2Impact microindentation studies in subjects with secondary osteoporosis.

**Table:** 

**(a) Endocrine disorders**										
**References**	**Subjects**	**(m/f)**	**Age (years)**	**FN BMD (g/cm**^**2**^**)**	**Fx prevalence (%)**	**BMSi**	**Relationship of BMSi with**			**Study quality scale (0–10)**
							**Age**	**BMD**	**Disease**	
Farr et al. (48)	Type 2 DM	30 (0/30)	65.4 (1.4)	0.93 (0.03)	10.0	77.2 (1.6) (73.9–80.6)	NA	NA	Lower in DM	5
	Controls	30 (0/30)	65.7 (1.6)	0.94 (0.03)	10.0	85.7 (1.6) (82.4–89.0)				
Nilsson et al. (41)	Type 2 DM	99 (0/99)	77.6 ± 1.5 (75–80)	0.69 ± 0.10	55.0	74.6 ± 7.6* (72.5–76.7)	NA	NA	Lower in DM	7
	Controls	954 (0/954)	77.7 ± 1.5 (75–80)	0.66 ± 0.10*	52.0	78.2 ± 7.5* (77.5–78.9)				
Furst et al. (49)	Type 2 DM	16 (0/16)	65.4 ± 2.4	0.77 ± 0.03	19.0	63.7 (1.9) (59.7 −67.8)	NA	No	Lower in DM	7
	Controls	19 (0/19)	65.6 ± 1.2	0.69 ± 0.01	11.0	70.1 (1.9) (66.1–74.1)				
Barnouin et al. (62) (Ab)	Type 2 DM	27 (NA)	NA (65–85)	NA	NA	70.5 ± 6.5 (67.9–73.1)	NA	NA	NA	NA
Holloway et al. (63) (Ab)	DM	34 (34/0)	NA (33–92)	0.97 (0.92–1.01)	NA	80.6 (78.9–82.9)	NA	NA	Lower in DM, but not in IFG	NA
	IFG	37 (37/0)		0.95 (0.91–0.99)		83.6 (81.7–85.6)				
	Controls	140 (140/0)		0.96 (0.94–0.98)		83.4 (82.4–84.4)				
Syversen et al. (64) (Ab)	Type 1 DM	33 (33/0)	42.7 ± 12.1	NA	NA	NA	NA	NA	Lower in DM	NA
	Controls	28 (28/0)	41.8 ± 12.0							
Malgo et al. (36)	Acromegaly	48 (26/22)	60.2 ± 11.0	0.84 ± 0.16	58.0	79.4 (0.7) (78.0–80.8)	Pos	No	Lower in Acromegaly	8
	Controls	44 (22/22)	60.5 ± 8.5	0.80 ± 0.09	16.0	83.2 (0.7) (81.8–84.6)	Neg	No		
Starr et al. (65) (Ab)	PHPT	13 (4/9)	59.3 ± 15.0	NA	NA	67.8 ± 9.0 (62.3–73.2)	NA	NA	Lower in PHPT + HypoPT	NA
	HypoPT	15 (4/11)	44.3 ± 12.5			68.4 ± 10.0 (62.9–73.9)				
	Controls	22 (5/17)	49.2 ± 17.0			77.2 ± 8.0 (73.7–80.7)				
**(b) HIV, Chronic kidney disease, CAG, MGUS**										
**CROSS-SECTIONAL DESIGN**										
Guerri-Fernandez et al. (46)	HIV	50 (35/15)	36.7 [31.7–46.2]	0.81 [0.77–0.88]	4.0	84.5 [83.0–87.0]	NA	NA	Lower in HIV	7
	Controls	35 (24/11)	33.9 [27.6–53.8]	0.79 [0.73–0.96]	0	90.0 [88.5–93.0]				
Guerri-Fernandez et al. (47)	HIV >5 years TDF/FTC	36 (27/9)	56.4 ± 6.3	0.72 [0.2]	5.5	81.0 [0.8]	No	No	Lower in TDF/FTC	7
	HIV >5 years ABC/3TC	27 (20/7)	63.0 ± 9.8	0.74 [0.2]	3.7	82.7 [1.3]				
Perez-Saez et al. (45)	ESRD before KT	53 (25/28)	55.8 ± 12.1	0.73 ± 0.15	26.4	79* [71.8–84.2]	NA	NA	Lower in ESRD	5
	Controls	94 (20/74)	50.2 ± 16.0	0.78 ± 0.12	0	82.6 [77.5–88.9]				
Perez-Saez et al. (58)	KT recipients >10 years after KT	40 (17/23)	63.8 ± 11.1	0.67 ± 0.13*	32.5	79.1 ± 7.7* (76.7–81.5)	NA	No	No	4
	Controls	94 (20/74)	50.2 ± 16.0	0.78 ± 0.12*	0	82.9 ± 7.8* (81.3–84.5)				
Aasarod et al. (56)	CAG	17 (9/8)	54.1 ± 12.6 (20–70)	0.79 ± 0.14	41.1	82.0 ± 9.6* (76.5–87.5)	NA	NA	No	1
	Controls	41 (20/21)	53.2 ± 11.4 (20–70)	0.80 ± 0.16	44.0	80.0 ± 7.0* (76.5–83.5)				
Gonzalez et al. (66) (Ab)	MGUS	22 (NA)	NA	0.73	NA	68.3 ± 5.0 (66.1–70.5)	NA	NA	Lower in MGUS	NA
	Controls	NA		0.78		83.0 ± 4.0

**Table:** 

**References**	**Intervention**	**Subjects**	**(m/f)**	**Age (years)**	**FN BMD (g/cm**^**2**^**)**	**Fx prevalence (%)**	**BMSi**	**Relationship of BMSi with**			**Study quality scale (0–10)**
								**Age**	**BMD**	**Intervention**	
**LONGITUDINAL DESIGN**											
Guerri-Fernandez et al. (52)	ART with TDF/FTC, FU 24 (not shown) + 48 weeks	HIV	40 (33/7)	38 ± 9	**BL:** 0.84 ± 0.12 **FU:** 0.81 ± 0.11	5.0	**BL:** 86.1 ± 6.1 (84.2–88.0) **FU:** 89.0 ± 4.2 (87.7–90.3)	Neg	Pos	Increase with ART	7
Lerma-Chippirraz et al. (59)	ART with TDF/FTC, FU 48 weeks (HIV only)	HIV	20 (16/4)	37 [31–43]	**BL:** 0.84 [0.79–1.02]**FU:** 0.82 [0.73–0.96]	0	**BL:** 86 [83–90]**FU:** 90 [88–93]	NA	NA	Increase with ART	4
		Controls	20 (15/5)	38 [35–42]	**BL:** 0.83 [0.75–0.98]	0	**BL:** 89 [88–93]				
Perez-Saez et al. (53)	Low-dose GC after KT, FU 3 (not shown) + 12 months	ESRD receiving KT	36 (19/17)	54.9 ± 11.6	**BL:** 0.75 ± 0.15 **FU:** 0.73 ± 0.14	16.7	**BL:** 79.2* [73.2–85.4] **FU:** 80.1* [73.0–85.4]	NA	NA	No	6

Age is presented as mean ± SD (range, if reported), mean (SE) or median [IQR], BMD is presented as mean ± SD, mean (95% CI), median [IQR] or median (SE) and BMSi is presented as mean ± SD (95% CI), mean (SE) (95% CI) or median [IQR]. 95% CI calculated from data provided in the publication. Ab, published only in Abstract form; ABC/3TC, Abacavir/lamivudine; ART, Antiretroviral therapy; BL, Baseline; BMD, Bone mineral density; BMSi, Bone material strength index; CAG, Chronic atrophic gastritis; ESRD, End-stage renal disease; FN, Femoral neck; FU, Follow-up; Fx, Fracture; IFG, Impaired fasting glucose; HypoPT, Hypoparathyroidism; MGUS, Monoclonal gammopathy of undetermined significance; PHPT, Primary hyperparathyroidism; DM, Diabetes mellitus; TDF/FTC, Tenofovir/emtricitabine. *Measured in a subgroup of subjects.

#### Endocrine Disorders

Secondary osteoporosis is a common co-morbidity of endocrine disorders, resulting from direct and indirect effects of hormonal excess or deficiency on the bone remodeling cycle, bone mineral content and bone matrix composition (71). The eight published studies in patients with endocrine disorders (four in abstract form) included a total of 304 patients in whom IMI was performed in addition to standard evaluation of fracture risk using DXA. Six of these studies were performed in patients with Diabetes mellitus (one in type 1, five in type 2) (41, 48, 49, 62–64), one in Acromegaly (36), and one in parathyroid disorders (primary hyperparathyroidism and hypoparathyroidism) (65).

##### Diabetes mellitus (DM)

The effect of DM on the skeleton is multifactorial. The main cause of osteoporosis in DM is low bone formation with the two main contributing factors for this being a shift from osteoblastogenesis to adipogenesis, and the toxic effects of the accumulation of advanced glycation end products (AGE) on osteoblasts, both leading to the characteristic low bone turnover and increased fracture risk particularly observed in type 1 DM (T1DM). Fracture risk is however increased in both T1DM and type 2 DM (T2DM) with BMD measurements shown to underestimate fracture risk (72). This suggests that tissue material properties of bone are likely to be impaired in patients with DM and that the evaluation of BMSi may provide information on bone strength not captured by BMD. Five of the six studies in DM patients (T1DM *n* = 33, T2DM *n* = 131), also including patients with impaired fasting glucose concentrations (IFG, *n* = 37), compared BMSi in DM and prediabetes patients with that of non-diabetic controls (*n* = 655). Findings from these studies show significantly lower BMSi values in all DM patients (type 1 and 2), but not in prediabetes, compared to controls, with values ranging from 63.7 (SE 1.9) (49) to 80.6 (63) in DM patients and from 70.1 (SE 1.9) (49) to 85.7 (SE 1.6) (48) in controls. Data from a US group also show an inverse relationship between BMSi and mean glycated hemoglobin level (HbA1c) over 10 years prior to the study (*r* = −0.41; *p* = 0.026) (48), suggesting a direct negative effect of prolonged hyperglycemia on bone material properties. This finding was also supported by a significant inverse relationship between BMSi and the duration of T2DM (*r* = −0.68, *p* < 0.05) observed in another US study (49). Data further show that BMSi is significantly inversely correlated with AGE data obtained from skin analysis as detected by autofluorescence (*r* = −0.65, *p* < 0.05) (49). In all these studies but one, LS and FN BMD were comparable between T2DM patients and controls, which was not the case in T1DM where a larger number of patients also had low bone mass. Data on fracture risk were not reported in any of the studies in patients with DM. Literature findings in DM therefore suggest that bone material properties are impaired in both T1DM and T2DM, independently of BMD. In the last of the six studies conducted in DM patients, BMSi was significantly higher in T2DM patients with higher ergonometrically measured fitness although this was only published in abstract form (62).

##### Acromegaly

Skeletal changes in acromegaly are due to GH excess and are characterized by high bone turnover in favor of increased bone formation. Although BMD is generally normal or increased, the disorder is associated with an increased risk for vertebral fractures (71). Our group showed that BMSi was significantly lower in 48 patients with acromegaly despite long-term remission: 16.1 years (range 0.5–37.8 years), compared with BMSi measurements in 44 age-matched controls, 79.4 (SE 0.7) vs. 83.2 (SE 0.7), *p* < 0.001, although LS and FN BMD were comparable between groups (36). This finding suggests that tissue material properties of bone are likely to be irreversibly altered in patients with acromegaly leading to persistent increased fracture risk despite long-term adequate control of GH excess (73). Intriguingly, we could not demonstrate a difference in BMSi between patients with (*n* = 28) or without (*n* = 20) vertebral fractures (36).

##### Parathyroid disorders

BMSi was measured in patients with parathyroid disorders in a single study only published in abstract form (65). Parathyroid hormone (PTH) plays an important role in the maintenance of bone mass and integrity, and both excess or decrease of the circulating hormone may potentially affect fracture risk. Whereas bone turnover is low in hypoparathyroidism, it is high in hyperparathyroidism in favor of bone resorption, resulting in bone loss particularly at cortical sites. Although both non-vertebral and vertebral fracture risk have been shown to be increased in hyperparathyroidism (74, 75), data in hypoparathyroidism are scarce and conflicting (76). Starr et al. found that BMSi was significantly lower in 13 patients with primary hyperparathyroidism than in 22 age- and sex-matched controls with normal PTH values (respectively, 67.8 ± 9.0 vs. 77.2 ± 8.0, *p* < 0.05). Interestingly, BMSi was also found to be lower in 15 patients with hypoparathyroidism compared to controls (respectively, 68.4 ± 10.0 vs. 77.2 ± 8.0, *p* < 0.05). As expected, BMD T-scores were higher in hypoparathyroidism than in hyperparathyroidism patients at all sites except at the LS. No data on fracture risk were provided (65).

#### Patients Infected With HIV

BMSi was measured in a total of 153 patients with HIV in four studies conducted by the Spanish group (46, 47, 52, 59). Patients infected with HIV are at increased fracture risk possibly due to the viral infection itself or its treatment, particularly when using the antiretroviral drug tenofovir disoproxil fumarate (TDF), although the exact mechanism by which this drug increases bone fragility remains elusive. In an initial study by Güerri-Fernández et al. BMSi was found to be significantly lower in 50 untreated HIV patients compared to 35 healthy HIV-negative controls [84.5 (83.0–87.0) vs. 90.0 (88.5–93.0), respectively, *p* < 0.001], in the presence of similar BMD values (46). Findings from two further studies showed a significant increase in BMSi from 86.1 ± 6.1 to 89.0 ± 4.2 (*p* < 0.001), reaching comparative values to those of healthy controls, 12 months after starting antiretroviral therapy with TDF, while BMD values decreased on treatment (52, 59). No data are provided on the effect of these findings on fracture risk. BMSi values were not found to be different in patients on long-term treatment with TDF compared to patients using the different antiretroviral agent abacavir, BMSi 81.0 (0.8) vs. 82.7 (1.3), *p* = 0.27, respectively (47).

#### Chronic Kidney Disease (CKD)

Data on BMSi in CKD were retrieved from three studies conducted by the Spanish group (45, 53, 58). BMSi values were significantly lower in 35 CKD patients on dialysis compared to 94 healthy non-CKD controls [respectively, 79.0 (71.8–84.2) vs. 82.6 (77.5–88.9), *p* < 0.05] (45). In a second study, BMSi was measured cross-sectionally in 38 kidney transplant recipients more than 10 years after kidney transplantation. BMSi was found to be low in long-term transplant recipients compared to 93 younger healthy controls, although the difference was no longer observed after adjusting for age, sex, and BMI. This suggests that bone material properties may improve in kidney transplant recipients in the long-term (58). The most recent data were from a longitudinal study conducted in 14 patients undergoing kidney transplantation on a low glucocorticoid dosing protocol with follow-up BMSi performed 3 and 12 months post-transplantation. These data showed no significant change in BMSi compared to baseline values both at 3 months and at 12 months post-transplant [respectively, 80.1 (73.0–85.4) vs. 79.2 (73.2–85.4), no *p*-value provided] despite a significant transient decrease of FN BMD at month 3 and a significant decrease of LS BMD at month 12 (53).

#### Chronic Atrophic Gastritis (CAG)

A small study in 14 Norwegian patients with CAG and 18 age- and sex-matched healthy controls reported no difference in BMSi between patients and controls (respectively, 82.0 ± 9.6 vs. 80.0 ± 7.0, no *p*-value provided) in the presence of similar LS and FN BMD values. No fracture data were provided in this study, and the only suggested potential contributory factor to fracture risk in this condition was the chronic gastric hypoacidity (56).

#### Monoclonal Gammopathy of Undetermined Significance (MGUS)

BMSi was measured in a single study only published in abstract form comparing data between 22 patients with MGUS and age-matched controls, the number of whom was not provided. Despite normal and comparable BMD values between MGUS patients and controls, a significantly lower BMSi was observed in MGUS patients compared to controls (respectively, 68.3 ± 5.0 vs. 83.0 ± 4.0, *p* < 0.001) (66). These findings suggest that impaired material properties of bone contribute to the increased fracture risk reported in MGUS (77).

### Impact Microindentation Studies in Rare Metabolic Bone Disorders (Table 3)

Three studies were performed in a total of 28 patients with rare metabolic bone disorders: One in Type 1 Gaucher Disease (*n* = 16) (57), one in Paget's disease of bone (*n* = 9) (67) and one in Camurati-Engelmann disease (*n* = 3) (60).

**Table 3:** Impact microindentation studies in subjects with rare metabolic bone disorders.

**References**	**Subjects**	**(m/f)**	**Age (years)**	**FN BMD (g/cm^**2**^)**	**Fx prevalence (%)**	**BMSi**	**Relationship of BMSi with**			**Study quality scale (0–10)**
							**Age**	**BMD**	**Disease**	
Herrera et al. (57)	Gaucher's disease	16 (7/9)	51.3 ± 14.4 (21–69)	NA	0.0	72.7 ± 10.0 (67.4–78.0)	NA	NA	Lower in Gaucher's disease	4
	Controls	29 (5/24)	48.7 ± 15.8 (20–69)		0.0	81.8 ± 1.4 (81.3–82.3)				
Malgo et al. (67)	Unilateral Paget's disease of the tibia	9 (4/5)	69.5 (55–87)	NA	NA	Pagetic Tibia: 74.7 (1.7) (70.8–78.6) Non-pagetic Tibia: 78.7 (1.3) (75.7–81.7)	NA	NA	NA	NA
	Controls	11 (7/4)	61.9 (51–72)	NA	72.7	Dominant Tibia: 82.1 (1.3) (79.2–85.0) Non-dominant Tibia: 81.4 (1.3) (78.5–84.3)				
Herrera et al. (60)	Camurati-Engelmann	3 (1/2)	44.0 [43–47]	NA	0.0	76.9	NA	NA	Lower in Camurati-Engelmann (ns)	2
	Controls	29 (5/24)	56.0 (NA)		0.0	81.4				

*Age is presented as mean ± SD (range), mean (range) or median [range] and BMSi is presented as mean ± SD (95% CI), mean (SE) (95% CI) or median. 95% CI calculated from data provided in the publication. BMD, Bone mineral density; BMSi, Bone material strength index; FN, Femoral neck; Fx, fracture*.

#### Type 1 Gaucher Disease

Type 1 Gaucher Disease is a rare lysosomal lipid storage disorder due to beta-glucocerebrosidase deficiency leading to the accumulation of glucocerebroside in cells of the macrophage lineage. In this disorder the mechanism of bone fragility is multifactorial including mechanical replacement of the bone marrow by direct infiltration by Gaucher cells and the increased production of inflammatory factors such as cytokines leading to an imbalance in bone turnover in favor of bone resorption (78). The lower BMSi values measured in 16 patients with type 1 Gaucher Disease compared to those of 29 age- and sex-matched healthy volunteers (respectively, 72.7 ± 10.0 vs. 81.8 ± 1.4, *p* < 0.05) are in keeping with the changes observed at predominantly cortical skeletal sites, although LS BMD was also decreased in these patients compared to controls. The main marker of disease activity, chitotriosidase, was also found to be inversely correlated with BMSi (*R*^2^ = 0.516, *p* < 0.05), suggesting that in Gaucher disease material properties of bone are more severely altered the more severe the disease is (57).

#### Paget's Disease of Bone

In a study performed by our group, BMSi values were measured in 9 patients with unilateral Paget's disease of the tibia, in remission after treatment with bisphosphonates. Data were compared with BMSi values of the contralateral non-pathologic tibia. We observed no significant difference in BMSi between affected and non-affected tibia, 74.7 (SE 1.7) vs. 78.7 (SE 1.3), *p* = 0.12. However, we did observe a significant difference in serial indentations of pathologic and normal tibia. The variation of consecutive single indentation values was significantly greater in the affected tibia compared to the contralateral healthy tibia, suggesting heterogenous tissue material properties of the pagetic bone. This high variability in measurements may represent a potential sign of altered material bone properties in Paget's disease of bone (67).

#### Camurati-Engelmann Disease

In a series of three patients with Camurati-Engelmann disease, a rare bone disease characterized by progressive hyperostosis mainly of the diaphysis of long bones, BMSi values were lower compared to BMSi values in 29 healthy controls, albeit non-significantly (76.9 vs. 81.4, *p* = 0.17), despite the characteristic cortical hyperostosis. This finding suggests that bone material properties can also be altered despite increased cortical volume (60).

### Impact Microindentation Studies in Patients Receiving Osteoporosis Treatment (Table 4)

BMSi was measured in three studies in patients receiving anti-osteoporotic therapy (*n* = 117) in order to evaluate whether IMI had the potential to be used in evaluating treatment-induced changes in bone fragility in osteoporosis (37, 44, 68).

Table 4Impact microindentation studies in subjects receiving osteoporosis treatment.

**Table:** 

**References**	**Subjects**	**(m/f)**	**Age (years)**	**FN BMD (g/cm^**2**^)**	**BMSi**	**Relationship of BMSi with**			**Study quality scale (0–10)**
						**Age**	**BMD**	**Fx**	
**CROSS-SECTIONAL DESIGN**									
Nogues et al. (37)	BP >4 years Fx	21 (0/21)	69.5 ± 5.9	0.62 ± 0.08	73.8 ± 6.5 (70.8–76.8)	NA	No	Pos	8
	BP > 4 years No Fx	19 (0/18)	71.5 ± 6.8	0.66 ± 0.09	81.6 ± 6.3 (78.5–84.7)

**Table:** 

**References**	**Intervention**	**OP treatment**	**(m/f)**	**Age (years)**	**FN BMD (g/cm**^**2**^**)**	**Fx prevalence (%)**	**BMSi**	**Relationship of BMSi with**			**Study quality scale (0–10)**
								**Age**	**BMD**	**Intervention**	
**LONGITUDINAL DESIGN**											
Mellibovsky et al. (44)	GC and OP prophylaxis, FU 7 and 20 weeks (not shown)	Ca/Vit D	19 (11/8)	55.3 ± 17.9	0.83 ± 0.13 FU NA	0.0	**BL:** 81.6 (74.3–86.9) **FU**: 71.9 (65.4–77.1)	NA	NA	Decrease in Ca/Vit DIncrease in TPTD + Dmab	7
		BP	14 (10/4)	66.1 ± 17.0	0.75 ± 0.14 FU NA	7.1	**BL:** 81.1 (75.6–89.6) **FU:** 83.4 (76.6–93.0)				
		TPTD	5 (1/4)	69.8 ± 8.0	0.62 ± 0.12 FU NA	60.0	**BL:** 70.0 (64.0–72.6) **FU:** 81.8 (73.3–88.9)				
		DMAb	14 (5/9)	58.9 ± 12.8	0.72 ± 0.15 FU NA	14.3	**BL:** 76.2 (72.0–84.9) **FU:** 84.0 (79.2–90.0)				
Tsai et al. (68) (Ab)	TPTD treatment, FU 3 months	TPTD 20 mg	33 (0/33)	NA(52–83)	NA	NA	**BL:** 82.1 ± 8.3* (61.5–102.7) **FU**: −4.8%	NA	NA	Decrease in TPTD 20 + 40 mg	NA
		TPTD 40 mg	29 (0/29)				**BL:** 83.2 ± 10.1* (67.1–99.3) **FU:** - 7.0%				

Age is presented as mean ± SD (range, if reported), BMD is presented as mean ± SD and BMSi is presented as mean ± SD (95% CI) or median (IQR). 95% CI calculated from data provided in the publication. Ab, published only in Abstract form; BL, Baseline; BMD, Bone mineral density; BMSi, Bone material strength index; BP, Bisphosphonate; Ca, Calcium; DMAb, Denosumab; FN, Femoral neck; FU, Follow-up; Fx, Fracture; GC, Glucocorticoid; OP, Osteoporosis; TPTD, Teriparatide. *Measured in a subgroup of subjects.

In the first study, BMSi was measured in 52 patients with various underlying diseases requiring glucocorticoid therapy before starting treatment with prophylactic anti-osteoporosis therapy. There was a significant decrease in BMSi after 7 weeks of treatment in the Calcium/Vitamin D3-treated group (*n* = 19), from 81.6 (74.3–86.9) to 71.9 (65.4–77.1) *p* < 0.05, compared to no significant changes in the Calcium/Vitamin D3 and additional oral bisphosphonate-treated group (*n* = 14), 81.1 (75.6–89.6) vs. 83.4 (76.6–93.0), *p* = 0.83. In contrast, a significant increase in BMSi [76.2 (72.0–84.9) to 84.0 (79.2–90.0), *p* < 0.05] was observed in the denosumab-treated group (*n* = 14), with the largest increase observed in a teriparatide-treated group (*n* = 5) [BMSi 70.0 (64.0–72.6) to 81.8 (73.3–88.9), *p* < 0.05]. It is of note that a significant increase of BMSi to 87.7 (78.7–96.5) was eventually also demonstrated in the oral bisphosphonate-treated group but only at the 20 weeks measurement timepoint compared to baseline (*p* = 0.043). This study was the first to show a change in BMSi in response to medical treatment (44).

In a second study published as a meeting abstract, BMSi was measured in seven patients with osteoporosis before and after 3 months of treatment with daily subcutaneous teriparatide 20 mcg (*n* = 3) or 40 mcg (*n* = 4). In this study a significant decrease in BMSi from baseline (−4.8 ± 10.7%, *p* = 0.011) was observed in the three patients receiving the lower dose of 20 mcg and the decrease was larger in the four patients receiving 40 mcg a day, −7.0 ± 15.5%, *p* = 0.011 (68). The Abstract format did not allow the authors to formulate a hypothesis for this discrepant finding. A full paper has not been published.

In the third study, BMSi was measured cross-sectionally in 39 patients with osteoporosis but without fractures before receiving bisphosphonate treatment for 4–14 years. BMSi values were found to be significantly lower in 21 patients with incident fractures under treatment with bisphosphonates, compared to 18 patients who did not sustain fractures during treatment (respectively, 73.8 ± 6.5 vs. 81.6 ± 6.3, *p* < 0.05). BMD at the LS was also lower in the 21 patients who had sustained incident fractures (0.66 ± 0.1 vs. 0.82 ± 0.1, *p* < 0.05) (37).

### Impact Microindentation Studies in Population-Based Cohorts (Table 5)

BMSi was measured in 489 individuals from a Swedish population-based cohort and in 357 subjects from an Australian cohort. The objective of the Swedish cohort, initiated in 2013, was to identify factors contributing to fracture risk in older women aged 75–80 years (39, 41, 43, 51). The Australian cohort was designed to investigate the epidemiology of osteoporosis in men across ages 33–96 years from the Geelong Osteoporosis study (54, 63, 69).

**Table 5:** Impact microindentation studies in population-based cohorts.

**References**	**Subjects**	**(m/f)**	**Age (years)**	**FN BMD (g/cm^**2**^)**	**Fx prevalence (%)**	**BMSi**	**Relationship of BMSi with**			**Study quality scale (0–10)**
							**Age**	**BMD**	**Fx**	
Rudang et al. (51)	Elderly women	211 (0/211)	78.3 ± 1.1 (75–80)	0.65 ± 0.10	55.5	75.6 ± 7.6 (74.6–76.6)	No	Pos	No	6
Sundh et al. (40)	Elderly women	202 (0/202)	78.2 ± 1.1 (75–80)	NA	NA	75.6 ± 7.6 (74.6–76.6)	NA	NA	No	8
Johansson et al. (43)	Elderly women VF	277 (0/277)	77.8 ± 1.4 (75–80)	0.64 ± 0.09*	100	76.9 ± 7.3* (75.7–78.1)	NA	NA	No	6
	Elderly women No VF	750 (0/750)	77.7 ± 1.6 (75–80)	0.67 ± 0.10*	0	77.9 ± 7.4* (77.1–78.7)				
Rufus-Membere et al. (69)	Men	252 (252/0)	63.2 ± 12.6 (33–96)	NA	NA	83.0 ± 6.4 (82.2–83.8)	No	NA	NA	NA
Rufus-Membere et al. (54)	Men	357 (357/0)	63.2 ± 13.8 (33–96)	0.96 ± 0.13	11.9	Fx 80.2 ± 6.9 (78.0–82.4) No Fx 82.8 ± 6.1 (82.1–83.5)	Neg	No	Pos	5

Age is presented as mean ± SD (range), BMD is presented as mean ± SD and BMSi is presented as mean ± SD (95% CI). 95% CI calculated from data provided in the publication. BMD, Bone mineral density; BMSi, Bone material strength index; FN, Femoral neck; Fx, Fracture; VF, Vertebral fracture. *Measured in a subgroup of subjects.

In the Swedish cohort, there was no difference in BMSi between fracture (*n* = 117) and non-fracture patients (*n* = 63), 76.1 ± 7.4 vs. 75.7 ± 7.9 (*p* = 0.4), also after stratification for low bone mass (51). BMSi did not also differ between women with vertebral fractures (*n* = 141) and those without (*n* = 331), 76.9 ± 7.3 vs. 77.9 ± 7.4, *p* = 0.15, nor was there an association between BMSi and number and/or severity of vertebral fractures (43). An inverse relationship was found between BMSi and the amount of subcutaneous fat at the tibia, whole body fat mass and BMI, suggesting a possible negative influence of adipose tissue on bone strength. BMSi was also found to be associated with cortical porosity as measured by HR-pQCT and cortical volumetric BMD at the distal tibia (39).

In a recent publication of data from the Australian cohort, analysis of the association between BMSi and FRAX clinical risk factors showed that BMSi was significantly lower in men with a prior fracture (*n* = 38), compared to those without (*n* = 319) (respectively, 80.2 ± 6.9 vs. 82.8 ± 6.1, *p* = 0.024), and in men with a history of parental hip fracture (*n* = 34) compared to those without (*n* = 323) (respectively, 80.1 ± 6.1 vs. 82.8 ± 6.9, *p* = 0.029). Data also showed that BMSi tended to be lower in the presence of T2DM (*n* = 44) and alcohol consumption (*n* = 60), albeit non-significantly, but not in the presence of smoking (*n* = 21) or secondary osteoporosis (*n* = 44) (54). A further study conducted in the Australian cohort addressed feasibility and tolerability of BMSi measurements in 252 consecutive individuals from the cohort. Data showed that the procedure was well accepted suggesting the potential promising use of this technique in the clinic as well as in research settings (69).

### Impact Microindentation Studies in Healthy Subjects (Table 6)

BMSi measurements were performed in healthy non-osteoporotic individuals (*n* = 206) in four studies. The first study focused on ethnical differences in BMSi measurements and was performed in 42 Norwegian women, aged 46.3 ± 13.6 years, and in 46 age-matched Spanish women, none of whom had ever sustained a fracture. BMSi was found to be significantly lower in Norwegian women compared to Spanish women (respectively, 77.0 ± 7.1 vs. 80.7 ± 7.8, *p* < 0.001), while total hip BMD was significantly higher in Norwegian than Spanish women (42). Differences in tissue-level material properties of bone might therefore partially explain geographically observed differences in fracture risk.

Table 6Impact microindentation studies in healthy subjects.

**Table:** 

**References**	**Subjects**	**(m/f)**	**Age (years)**	**FN BMD (g/cm^**2**^)**	**Fx prevalence (%)**	**BMSi**	**Relationship of BMSi with**			**Study quality scale (0−10)**
							**Age**	**BMD**	**Fx**	
**CROSS-SECTIONAL DESIGN**										
Duarte Sosa et al. (42)	Women from Norway	42 (0/42)	46.3 ± 13.6	1.03 ± 0.10	0	77.0 ± 7.1 (74.9–79.2)	No	No	NA	5
	Women from Spain	46 (0/46)	46.7 ± 15.4	0.83 ± 0.12	0	80.7 ± 7.8 (78.4–83.0)				
Taymouri et al. (61)	Healthy volunteers	88 (19/69)	Men: 34 (24–98) Women: 49 (30–81)	NA	NA	88.0 ± 7.6 (84.3–91.7) 82.0 ± 7.4 (80.3–83.8)	No	NA	NA	3
Guerri et al. (70) (Ab)	Subjects prior to knee replacement	10 (5/5)	72 ± 5 (59–83)	0.66 ± 0.08	NA	75.8 ± 6.0 (71.5–80.1)	NA	No	NA	NA

**Table:** 

**References**	**Intervention**	**Subjects**	**(m/f)**	**Age (years)**	**FN BMD (g/cm**^**2**^**)**	**Fx prevalence (%)**	**BMSi**	**Relationship of BMSi with**			**Study quality scale (0−10)**
								**Age**	**BMD**	**Fx**	
**LONGITUDINAL DESIGN**											
Sundh et al. (40)	Exercise of one leg, FU after 3 months	Inactive women	20 (0/20)	55.5 ± 2.3 (51–59)	**BL:** 0.72 ± 0.08 **FU:** NA	NA	**BL:** 73.4 ± 5.8 (70.7–76.1) **FU:** 76.8 ± 9.0 (72.6– 81.0)	NA	NA	NA	8

*Age is presented as mean ± SD (range, if reported) or median (range), BMD is presented as mean ± SD and BMSi is presented as mean ± SD (95% CI). 95% CI calculated from data provided in the publication. Ab, published only in Abstract form; BL, Baseline; BMD, Bone mineral density; BMSi, Bone material strength index; FN, Femoral neck; FU, Follow-up; Fx, Fracture*.

BMSi was sequentially measured in 20 healthy non-osteoporotic postmenopausal women, aged 51–60 years, before and 3 months after starting a unilateral high impact exercise program. Findings from this study showed an increase in BMSi of 7% from 73.4 ± 5.8 to 76.8 ± 9.0 (*p* = 0.03) in the exercised leg, without concomitant changes in volumetric BMD or bone microarchitecture. The authors concluded that sequential IMI may detect improvement in bone material properties within 3 months of high-impact loading before changes in bone mass or architecture can be detected (40).

Two further studies performed in non-osteoporotic individuals specifically investigated the relationship between BMSi and age in 69 women with a median age of 49 years (range 30–81 years), and 19 men with a median age of 34 years (range 24–98 years) (61), and the relationship between BMSi and BMD in 5 men and 5 women, aged 72 ± 5 years (59–83 years) (70). Data from these studies show that neither age nor BMD were associated with BMSi in non-osteoporotic individuals.

### Factors Influencing BMSi

The systematic review of the literature on IMI performed for any indication revealed variable outcomes with the use of this tool depending not only on subject- or disease-related factors such as age, gender, BMI, geographical region or prevalent fractures and BMD, but also depending on technique-related factors such as experience and number of operators per study, number of indentations obtained per BMSi measured, and strategies used to assess inadequate quality of single indentations.

#### Subject-Related Factors

##### Age

Published data on the relationship between BMSi and age are conflicting. Whereas, eight studies failed to show a significant relationship between these two parameters (35, 38, 42, 47, 51, 55, 61, 69), six studies did demonstrate an inverse relationship between the two (33, 34, 36, 50, 52, 54) and only one study performed in 48 patients with acromegaly, aged 60.2 ± 11.0 years, showed a significant positive relationship between BMSi and age (*r* = 0.291, *p* = 0.045) (36).

Four of the eight studies showing no significant correlation between BMSi and age included subjects with very low BMSi values (35, 38, 42, 51) or a narrow age range (75–80 years) (51). A fifth and a sixth study included heterogenous groups of postmenopausal women with atypical femoral fractures (*n* = 20), hip fractures (*n* = 15), long-term bisphosphonate use (*n* = 30), and treatment-naive controls (*n* = 88) (55), or two groups of HIV-infected patients on long-term treatment with different types of antiretroviral agents (47). A relationship between BMSi and age could not be observed in a study designed to address this issue conducted in 88 patients: 69 women aged 49 years (range 30–81 years), and 19 men aged 34 years (range 24–98 years) (61). The last study that failed to show a relationship between BMSi and age was performed in the Australian cohort, which included 252 men with a wide age range: 33–96 years (69). However, after increasing the sample size of the cohort to 357 subjects, an inverse relationship between BMSi and age did become apparent (*r* = −0.13, *p* = 0.014) (54).

Among the five other studies showing an inverse relationship between BMSi and age, three were from our group and included a total of 164 subjects, aged 61.8 ± 9.4 years (range 40–85 years; *r* = −0.457, *p* = 0.002), all of whom were evaluated for increased fracture risk (33, 34, 36). The two other studies reported similar results in 191 postmenopausal women older than 50 years of age (*r* = −0.15, *p* = 0.03) (50), albeit non-significant in 40 HIV-positive subjects, aged 38 ± 9 years (*r* = −0.28, *p* = 0.07) (52).

##### Gender

Six studies have directly compared BMSi values between men (*n* = 208) and women (*n* = 186) (33, 34, 36, 46, 47, 56), one of which was conducted in HIV-infected patients. A significantly higher BMSi was observed in men (*n* = 35) compared to women (*n* = 15), 85.0 [83–87] vs. 80.0 (77–83), *p* < 0.001. In the same study there was no difference observed in 24 HIV-negative men and 11 HIV-negative women, BMSi 92.0 [88–96] vs. 89.0 [86–93], *p* = 0.07 (46). There was also no gender difference in the other five studies comparing BMSi values in men and women (33, 34, 36, 47, 56).

##### BMI

Data from three studies show BMSi to be significantly inversely correlated with BMI (*n* = 559) (39, 54, 69), although this association could not be confirmed in four other studies (*n* = 365) (33, 34, 36, 55).

##### Geographical variation

One study specifically addressed geographical variation in BMSi and significant differences were observed between different countries, with healthy Norwegian women having lower BMSi than healthy Spanish women (42). No other study addressed geographical variation in BMSi.

#### Fracture-Related Factors

##### BMD and fractures

Six studies reported significantly lower BMD values at one or more sites in patients with fractures compared to those without (35, 37, 38, 43, 50, 51). One study found a relationship between BMD and hip fractures but not atypical femoral fractures (55), while five studies found no significant difference in BMD between patients with or without fractures (33, 34, 45, 54, 58). Whereas the majority of reported studies (*n* = 14) did not elicit a significant association between BMSi values and BMD measurements (33–38, 42, 45, 47, 49, 54, 55, 58, 70), three did observe a weak correlation between the two measurements (50–52).

##### BMSi and fractures

Six studies found significantly lower BMSi values in patients with low bone mass and fragility or stress fractures, compared to non-fracture controls (33–35, 37, 38, 54). One study reported a significant relationship between BMSi and distal radius fractures, but not hip fractures (50). However, seven other studies did not observe a significant relationship between BMSi and fractures. These were three studies in patients with a diagnosis other than osteoporosis (36, 45, 58), three epidemiological studies from the Swedish cohort including patients with fractures irrespective of trauma type (39, 43, 51), and a study including 15 patients with the rare atypical femoral fractures and 20 patients with hip fractures (55).

#### Technique-Related Factors

As mentioned above, variability of data collected might also be due to technique-related factors such as experience of the operator, number of operators performing the technique per study, number of indentations obtained per BMSi evaluation and strategies used to assess false single indentations.

##### Operator-related factors

Literature data about operator training and experience are scarce and only provided in eight of the 38 publications yielded by our search (33, 43, 48, 50, 52, 54, 61, 69). Data on intra-observer coefficient of variation (CV) provided in 16 of the 38 publications (33–36, 38–43, 46, 48, 49, 51, 52, 67) report variations ranging from 1.65% (48) to 9.1% (42). Nineteen studies provided information about the number of operators performing the IMI measurements, ranging from one to nine different operators per study (33–35, 37–40, 42–44, 49–52, 54, 55, 61, 67, 69). Data on interobserver variability were however scarce, only reported in five of the 19 studies (39, 40, 43, 51, 61), with four of the five studies being from the same group reporting an interobserver CV of 5.2% for which data were adjusted before analysis (39, 40, 43, 51). The fifth study reported an interobserver CV of “<5%” (61).

##### Measurement-related factors

BMSi, the outcome parameter of IMI, is a dimensionless value calculated by the OsteoProbe® software from the mean of repeated indentations on the tibia, normalized to the mean of indentations performed on the phantom PMMA material. Although 13 studies (37, 45, 47, 50, 52–55, 57, 60, 61, 67, 69) stated that they performed the IMI measurements according to the standard operating procedure published in 2016 (8), the number of indentations obtained on the tibia, which was provided by all 31 full publications, varied widely from 5 up to 25 indentations. In contrast, the number of indentations performed on PMMA material provided by the majority of studies (*n* = 28) was consistently five as per standard protocol (33–35, 37–41, 43–55, 57–61, 67, 69). Since the older software does not automatically flag inadequate indentations, the evaluation of the adequacy of an indentation was entirely left to the judgement of the operator. Data on how inadequate indentations were identified, and methods used to delete these indentations, were not available for seven of the full publications (39, 42, 43, 48, 56, 58) and when reported, differed between studies (33–38, 40, 41, 44–47, 49–55, 57, 59–61, 67, 69).

### Tolerability and Safety

Fourteen publications reported that the microindentation investigation using the OsteoProbe® was well tolerated and not associated with any major complications (35, 37, 38, 42, 44, 46–48, 51, 52, 54, 57, 60, 69). Only two minor complications were reported, a mild skin infection and a mild allergic reaction to the local anesthetic, both of which readily responded to treatment.

## Discussion

Identifying the patient at increased risk for a fragility fracture may be challenging, particularly in patients with secondary factors for osteoporosis, as bone mineral density measurements using DXA have been shown to underestimate true fracture risk in these patients (71, 72, 79–81). Over the past decade, evidence has been accumulating about the value of impact microindentation in the *in vivo* assessment of tissue-level material properties of bone, an important contributor to bone strength in addition to that of BMD. The number of studies addressing the value of bone material strength index (BMSi) measurements in the evaluation of fracture risk has been steadily increasing, but outcomes are not always concordant. The main aim of this systematic review of the literature was to examine the added value of impact microindentation in the evaluation of fracture risk in clinical practice.

Our search yielded 38 studies that were published over the past 5 years including 19 studies published in the first 3 years and reported in a previous review (9). Data from these 38 studies highlight the ability of IMI in identifying patients with increased bone fragility, be it patients with primary osteoporosis and fractures, or patients with or at risk for secondary osteoporosis due to a variety of underlying systemic disorders including endocrine disorders such as diabetes mellitus or acromegaly. Data on the value of IMI in the follow-up of patients with increased fracture risk remain scarce but do suggest that the technique is also able to detect changes in BMSi following bone-modifying therapy in both in primary and secondary osteoporosis. The scarce data published in patients with rare metabolic bone disorders such as Type 1 Gaucher Disease or Camurati-Engelmann disease also provide valuable insights into the relationship between tissue-level material properties of bone and fracture risk.

The evaluation of BMSi is of particular interest in patients who have sustained a fragility fracture in the presence of osteopenia or normal BMD values, where DXA BMD measurements underestimate fracture risk. More than 50% of fragility fractures have been found to occur in patients with osteopenia (1), the majority of whom are currently not being offered treatment with bone-modifying agents according to most nationwide adopted treatment protocols. Studies in primary osteoporosis show that IMI could identify patients with fragility fractures, also in the subgroup of those with osteopenia (33, 38), suggesting that tissue material properties of bone are altered in patients who have sustained a fragility fracture and that IMI might therefore help in identifying patients with primary osteoporosis at increased fracture risk where DXA fails to do so. Although BMSi is measured at a cortical site, most studies are concordant in showing that low BMSi is associated with increased bone fragility at all relevant skeletal sites, vertebral, non-vertebral and hip sites (34, 35, 50). Studies that did not elicit a relationship between BMSi and fractures were either performed in small subgroups of patients with secondary osteoporosis (36, 45, 58), or in studies including patients with the lowest reported BMD values among all studies (43, 51). These findings suggest that BMSi may not be of added value in evaluating bone fragility in the presence of severely decreased bone mass which is highly predictive of high fracture risk. On the other hand, findings also suggest that in patients with very low BMSi values and high fracture risk due to impaired tissue material properties of bone, bone mass may be of less important contribution to bone fragility, as observed in studies investigating BMSi and BMD in type 2 diabetes mellitus patients (41, 48, 63). In keeping with this hypothesis, BMSi was indeed found to be low in almost all studies including patient groups with a variety of underlying systemic disorders associated with secondary osteoporosis, where bone quality rather than bone quantity is likely to have the most impact on fracture risk. It is of note that BMSi was also found to be inversely correlated with markers of underlying disease activity in secondary osteoporosis such as AGE accumulation in type 2 diabetes mellitus (49), and serum chitotriosidase levels in type 1 Gaucher Disease (57), suggesting that material properties of bone are more severely altered the more severely uncontrolled the disease is. Since BMSi values have been found to be independent of BMD values in almost all studies, findings from this systematic review strongly suggest that IMI captures elements of bone fragility not captured by BMD measurements. However, although IMI measurements have been shown to identify patients at increased risk for fracture, it has not fully been elucidated which specific mechanical properties of bone are captured by IMI. A recent study from our group addresses this issue for the first time by simultaneously evaluating tissue material properties of bone by measuring BMSi and bone composition of trans-iliac bone biopsies in humans *in vivo* in 12 patients with a variety of metabolic bone disorders and variable fracture risk (five patients with osteopenia and fractures, four patients with secondary osteoporosis, and three patients with rare bone diseases). The demonstration of both a negative correlation between BMSi and cortical porosity at the micro- and nanolevel and of a positive correlation of BMSi with the bone organic matrix parameters glycosaminoglycan and pyridinoline, and with mineral parameters, suggests that BMSi is affected by the composition of bone organic matrix and by bone mineral properties (82). The IMI technique has therefore the potential to be used as an additional tool to DXA in the evaluation, and also probably follow-up, of bone fragility in the clinic since sequential BMSi measurements have been shown to increase in situations associated with a decrease in fracture risk (44, 52, 59), and to decline where fracture risk increases, such as observed in patients starting glucocorticoids (44).

Notwithstanding, this systematic review of the literature encountered a number of limitations in the interpretation of the generated data. One of these limitations was the high variability in the methodological quality of the published studies. Although on the whole study quality was judged to be satisfactory, quality scores ranged widely from 1 to 10 out of 10 possible points using the adapted Newcastle-Ottawa Scale. Most studies included small numbers of patients and were performed in selected patient groups, potentially resulting in a selection bias. It should also be emphasized that none of the published studies on IMI had longitudinal fracture data and the majority had a cross-sectional design except for six studies with sequential BMSi measurements under an intervention with either bone-modifying agents or exercise. Although low BMSi as measured by IMI has been clearly linked with the presence of any type of fracture, the limitations attached to a number of the studies addressing this issue do not permit, so far, to extrapolate that BMSi measurements may be predictive for increased future fracture risk. In addition, although tissue material properties of bone as measured by IMI have been shown to be altered in almost all studies investigating patient groups with secondary osteoporosis, only three of these studies compared BMSi values of fractured patients with those of non-fractured ones. No significant difference in BMSi values was observed between patients with and without fractures, although subgroups were small.

Another important limitation encountered in the interpretation of IMI data generated by our systematic literature review is the high variability of BMSi values observed in the control arm subjects between studies. Some studies reported BMSi values of 81 or higher in control subjects, whilst others reported lower BMSi values in the range of 70–78, corresponding to the range of BMSi values observed in patient groups with increased fracture risk; Tables 1–6.

Our systematic review has identified a number of potential subject-, disease- and technique-related factors for this discrepancy. Our and other groups consistently reported a significant relationship between BMSi and age, with a significant decrease in BMSi observed with increasing age. This indicates that tissue material properties of bone as measured with IMI do decline with age, which would also be expected given the increasing fracture risk associated with aging. The absence of an observed relationship between BMSi and age in some studies may possibly be due to a narrow age range (46, 51), a small number of patients included (61), or the inclusion of heterogenous groups of patients (55). In addition, studies that did not observe a relationship between BMSi and age report some of the lowest BMSi values measured (35, 38, 42, 51). Geographical differences might also influence BMSi values as observed in a study specifically designed to address this issue (42), and this is supported by the observation of this review that non-fractured elderly women from Sweden had BMSi values below 78 (39, 43, 51), which were remarkably lower than BMSi values of non-fractured controls from The Netherlands (33, 34) and Spain, who had BMSi values above 81 (37, 45, 46, 57, 58, 60, 61). Different BMSi values of subjects from different geographical regions could reflect their difference in fracture risk and this should be taken into account when comparing studies. The only currently available age-dependent reference values are those suggested by the manufacturer (Active Life Scientific) and these remain to be confirmed.

Another complicating issue in the interpretation of BMSi results is that currently used IMI protocols vary in a number of key points, which hampers pooling of data and general application of the technique and may partly explain the variation in BMSi outcome between studies. The OsteoProbe® is a relatively easy to use handheld device specifically designed for *in vivo* use in humans, but it is also prone to variability in its results, which is at least partly due to the lack of a standard operating procedure until the recently published technical recommendations by Diez-Perez et al. (8). However, studies still use different protocols even after this publication. Part of the identified technique-related aspects of IMI will be addressed by the introduction of an updated system in which the software will automatically discard measurements that are more than two standard deviations away from the subject's mean, although large deviations might reflect bone disease (67). Of additional importance is adequate operator training and limitation of the number of operators per study, to minimize intra- and interobserver variability. Only after these methodological inconsistencies are addressed and future measurements are strictly conducted according to the standard protocol can normative values be obtained, and data compared between studies and centers.

Taken together, findings from this systematic review of the literature show that impact microindentation is a promising technique enabling physicians to evaluate *in vivo* tissue-level material properties of bone in a minimally invasive, simple and safe manner. Data generated by impact microindentation have contributed to the better understanding of factors involved in the pathogenesis of bone fragility. Data have also been shown to be valuable not only in the evaluation of bone fragility, but possibly also in the follow-up, particularly of patients with potentially underestimated fracture risk. BMSi is not a measure of bone mass, and no clear relationship has been demonstrated between the two, implying that IMI could be used as an additional tool to DXA BMD in the assessment of bone health rather than as a replacement of the latter in the individual patient. However, the value of IMI in predicting future fractures and treatment outcomes has yet to be established in prospective multicenter trials using standard operating procedures before recommending the routine use of the technique in the clinic.

## Author Contributions

MS, NH, and NA-D: conception of the work. MS and NA-D: data collection, data analysis, and interpretation. MS, NH, FM, and NA-D: drafting the article. MS, NH, FM, EW, and NA-D: critical revision of the article and final approval of the version to be published.

### Conflict of Interest

NA-D is an unpaid member of the Scientific Board of Active Life Scientific, manufacturer of the OsteoProbe®. The remaining authors declare that the research was conducted in the absence of any commercial or financial relationships that could be construed as a potential conflict of interest.
