# Supplementary material for: Added Value of Impact Microindentation in the Evaluation of Bone Fragility: A Systematic Review of the Literature
Source: Front Endocrinol (Lausanne). 2020 Feb 7;11:15. doi: 10.3389/fendo.2020.00015 (PMC7020781; doi:10.3389/fendo.2020.00015)
Supplement: Supplementary file 1 [file Data_Sheet_1.pdf]

Supplementary Material

1. Search strategy

Pubmed

(("reference point indentation"[tw] OR (("indentation"[tw OR "microindentation"[tw] OR indentat*[tw] OR microindentat*[tw]) AND ("reference point"[tw] OR "reference points"[tw] OR reference point*[tw])) OR (("indentation"[tw] OR "microindentation"[tw] OR indentat*[tw] OR microindentat*[tw]) AND ("Bone Density"[mesh] OR "bone density"[tw] OR "bone mineral density"[tw] OR "bone fragility"[tw])) OR "impact microindentation"[tw] OR "Osteoprobe"[tw] OR osteoprob*[tw] OR ("Bone Material"[tw] AND ("Clinical Risk Factor"[tw] OR "Clinical Risk Factors"[tw]) AND Fractur*[tw])) NOT ("Animals"[mesh] NOT "Humans"[mesh]))

Embase

(("reference point indentation".mp OR (("indentation".mp OR "microindentation".mp OR indentat*.mp OR microindentat*.mp) AND ("reference point".mp OR "reference points".mp OR reference point*.mp)) OR (("indentation".mp OR "microindentation".mp OR indentat*.mp OR microindentat*.mp) AND (exp "Bone Density"/ OR "bone density".mp OR "bone mineral density".mp OR "Bone Fragility"/ OR "bone fragility".mp)) OR "impact microindentation".mp OR "Osteoprobe".mp OR osteoprob*.mp OR ("Bone Material".mp AND ("Clinical Risk Factor".mp OR "Clinical Risk Factors".mp) AND Fractur*.mp)) AND exp "Humans"/)

Web of Science

(ts=("reference point indentation" OR (("indentation" OR "microindentation" OR indentat* OR microindentat*) AND ("reference point" OR "reference points" OR "reference point*")) OR (("indentation" OR "microindentation" OR indentat* OR microindentat*) AND ("Bone Density" OR "bone density" OR "bone mineral density" OR "Bone Fragility" OR "bone fragility")) OR "impact microindentation" OR "Osteoprobe" OR osteoprob* OR ("Bone Material" AND "Clinical Risk Factor*" AND Fractur*)) NOT ti=(veterinary OR rabbit OR rabbits OR animal OR animals OR mouse OR mice OR rodent OR rodents OR rat OR rats OR pig OR pigs OR porcine OR horse* OR equine OR cow OR cows OR bovine OR goat OR goats OR sheep OR ovine OR canine OR dog OR dogs OR feline OR cat OR cats))

2. Supplementary Table

Supplementary Table. Adapted Newcastle Ottawa Scale

This scale has been adapted from the Newcastle-Ottawa Quality Assessment Scale to provide a quality assessment.

Selection: (Maximum 3 points)

1. Representativeness of the sample:
    a. Obviously representative of the average in the target population (all subjects or random sampling). *
    b. Somewhat representative of the average in the target group (non-random sampling). *
    c. Convenience sample.
    d. No description of the derivation of the included subjects.

2. Sample size:
    a. Justified, including sample size calculation. *
    b. Not justified.
    c. No information provided.

3. Non-respondents:
    a. Proportion of target sample recruited attains pre-specified target and basic summary of non-respondent characteristics in sampling frame recorded. *
    b. Unsatisfactory recruitment rate or no summary data on non-respondents.
    c. No information provided.

Comparability: (Maximum 2 points)

1. Comparability of subjects in different outcome groups on the basis of design or analysis. Confounding factors controlled.
    a. Data/ results adjusted for relevant confounders, including age (if applicable) and operator (if multiple). **
    b. Only adjusted for relevant confounders, excluding operator / no information regarding number of operators. *
    c. Data/results not adjusted for relevant confounders / no information regarding confounders or number of operators.

Exposure/Outcome: (Maximum 5 points)

1. Application of the Impact Microindentation technique
   a. Information if certain measurements are flagged and intraobserver CV provided. **
   b. Only one of the two under a. provided information is given. *
   c. No description of flagged measurements and intraobserver CV.

2. Application of Vertebral fracture assessment tools
   a. Use of vertebral fracture assessment or spinal radiographs for detecting VF. **
   b. Medical records. *
   c. Vertebral fractures only self-reported.

3. Statistical test:
   a. The statistical test used to analyze the data is clearly described and appropriate, and the measurement of the association is presented (including SD/SE and the probability level; p value). *
   b. The statistical test is not appropriate, not described or incomplete.

Good: 8-10 points

Satisfactory: 5-7 points

Unsatisfactory: 0 to 4 points
